# Corrosion Inhibitor-Modified Plasma Electrolytic Oxidation Coatings on 6061 Aluminum Alloy

**DOI:** 10.3390/ma14030619

**Published:** 2021-01-29

**Authors:** Maciej Sowa, Marta Wala, Agata Blacha-Grzechnik, Artur Maciej, Alicja Kazek-Kęsik, Agnieszka Stolarczyk, Wojciech Simka

**Affiliations:** Faculty of Chemistry, Silesian University of Technology, B. Krzywoustego Street 6, 44-100 Gliwice, Poland; marta.wala@polsl.pl (M.W.); agata.blacha-grzechnik@polsl.pl (A.B.-G.); artur.maciej@polsl.pl (A.M.); alicja.kazek-kesik@polsl.pl (A.K.-K.); agnieszka.stolarczyk@polsl.pl (A.S.)

**Keywords:** plasma electrolytic oxidation, corrosion resistance, corrosion inhibitor, 8-hydroxyquinoline, X-ray diffraction, X-ray photoelectron spectroscopy, Raman spectroscopy, aluminum alloy

## Abstract

There are many methods for incorporating organic corrosion inhibitors to oxide coatings formed on aluminum alloys. However, typically they require relatively concentrated solutions of inhibitors, possibly generating a problematic waste and/or are time-/energy-consuming (elevated temperature is usually needed). The authors propose a three-step method of oxide layer formation on 6061-T651 aluminum alloy (AAs) via alternating current (AC) plasma electrolytic oxidation (PEO), impregnation with an 8-hydroxyquinoline (8-HQ) solution, and final sealing by an additional direct current (DC) polarization in the original PEO electrolyte. The obtained coatings were characterized by scanning electron microscopy, roughness tests, contact angle measurements, X-ray diffraction, Raman spectroscopy, and X-ray photoelectron spectroscopy. Additionally, corrosion resistance was assessed by potentiodynamic polarization in a NaCl solution. Two types of the coating were formed (A—thicker, more porous at 440 mA cm^−2^; B—thinner, more compact at 220 mA cm^−2^) on the AA substrate. The 8-HQ impregnation was successful as evidenced by XPS. It increased the contact angle only for the B coatings and improved the corrosion resistance of both coating systems. Additional DC treatment destroyed superficially adsorbed 8-HQ. However, it served to block the coating pores (contact angle ≈ 80°) which improved the corrosion resistance of the coating systems. DC sealing alone did not bring about the same anti-corrosion properties as the combined 8-HQ impregnation and DC treatment which dispels the notion that the provision of the inhibitor was a needless step in the procedure. The proposed method of AA surface treatment suffered from unsatisfactory uniformity of the sealing for the thicker coatings, which needs to be amended in future efforts for optimization of the procedure.

## 1. Introduction

Despite its advantages, such as low density and good resistance towards atmospheric corrosion, pure aluminum is rarely used as a construction material because of its high plasticity and softness. To amend these problems, aluminum is alloyed with other elements, such as copper, magnesium, zinc, or silicon to improve the features of the final material. Unfortunately, materials containing aluminum as the main metallic element, even after alloying with other metals, show sensitivity towards pitting corrosion. Pitting is one of the most dangerous types of corrosion because it can show almost no external signs of damage on the material surface until its destruction [1,2].

To prevent pitting a few methods of surface modification can be used. One of the most popular ones is anodizing—the process of covering the surface of a protected element with an oxide layer. The aluminum oxide layer produced in such a manner is composed of columnar nanometric pores on top of a thin barrier sublayer, and to fully protect an element from the corrosion a process of additional sealing of the obtained coating is necessary [3]. Plasma electrolytic oxidation (PEO) is an extension of the anodizing process because (similarly to the anodizing) it consists of the anodic polarization of a workpiece. However, the forming potentials utilized in the case of PEO are high enough to exceed the potential of oxide dielectric breakdown (giving rise to plasma formation). Consequently, thick, hard, and micro-porous oxide layers can be obtained. Moreover, the PEO layers are notorious for their excellent adherence to the substrate material, even if the workpiece exhibits complex geometry [4,5,6]. Additionally, because of relatively high growth rates encountered in the process [7] combined with the unusual mechanism of oxide formation constituting plasma formation and quenching [4,5,8], electrolytic bath ingredients can be incorporated into the layer allowing for easy modification of the coating composition [1,3].

If plasma electrolytic oxidation of aluminum or its alloys is carried out under an AC regime “soft sparking” phenomenon can take place. Symptoms such as a sudden drop of the cell voltage, lowering of the intensity of sparking, and silencing of acoustic emissions accompanying the process are all hallmarks of the “soft-sparking” regime [5,9]. It is postulated that this phenomenon is probably caused by the growth of the oxide layer to the point when the dielectric breakdown is no longer capable of passing throughout the whole thickness of the coating, but rather takes place in its inner part. This leads to impeded heat exchange from the coating to the electrolyte and the changes in the internal phase composition of the formed oxide layer. As a result, relatively smoother, less porous, and thicker oxide layers, enriched in α-alumina can be obtained on the Al-based substrates [1,9,10]. The prerequisite necessary for attaining “soft sparking” during AC PEO is the correct modulation of imbalance between positive and negative charges passed through the treated metal surface. For this purpose, the *R*_Q_ parameter, describing the ratio of the positive to the negative charges in the AC signal of the PEO, has been introduced. Practice shows that the values of *R*_Q_ less than 1 are conducive for entering the “soft-sparking” regime [5,9,10].

The composition of PEO coatings can be modified not only by changing the process conditions but also in the course of various post-treatment strategies. Because of the high porosity of the formed oxide layers, the incorporation of chemical substances by their impregnation into the sponge-like oxide is readily possible. In this way, many surface features, such as hydrophobicity [11,12], corrosion protection [13,14,15], or self-lubricating properties [16] can be attained by the final product. Some attempts have been made at incorporating layered double hydroxide (LDH) structures into the PEO porous oxide layers. The LDH themselves, because of their superior ion exchange capabilities, were then treated as nanocontainers for a load of selected corrosion inhibitors of aluminum, such as vanadate ions [13] or phytic acid [15].

When it comes down to the improvement of the corrosion resistance of the aluminum alloys, researchers have tested a few possible ways of modifying PEO oxide surfaces with corrosion inhibitors, such as impregnation of the coating by appropriate solutions such substances [17,18] which might be followed by dipping in an epoxy resin [19]. Furthermore, the incorporation of zeolite particles loaded with corrosion-inhibiting cerium ions into the PEO electrolytic bath was also exercised to give rise to a highly protective surface layer on an AZ31 magnesium alloy [20]. Many other approaches were tested, from the deposition of silica skeletons onto the surface of a PEO layer and their doping with an inhibitor solution [12], through loading of the PEO layer by immersion in a sodium hydroxide solution of inhibitor followed by drying at an elevated temperature [21], to the immersion of PEO layers in the solutions containing corrosion inhibitors at elevated temperature [14].

In this work, a different approach to the problem of boosting the corrosion resistance of the PEO-ed aluminum alloys is presented. First, highly porous and hydrophilic oxide layers have been obtained in an AC PEO process with the inclusion of the “soft sparking” phenomenon. As the surface plasma events occurring during this phase of the process are less intense, the authors wanted to see if it would be possible to take advantage of the milder conditions and incorporate organic corrosion inhibitor species into the growing oxide. In such a scenario this substance could become sealed in the coating, without the need for time- and energy-consuming post-treatment which additionally generates a troublesome waste (e.g., concentrated inhibitor solutions). To this end, the authors have adopted a three-step procedure of formation of the oxide layer on a 6061-T651 aluminum alloy substrate under the AC PEO conditions, impregnation of the oxide with an ethanolic solution of a selected corrosion inhibitor—8-hydroxyquinoline (8-HQ), and final sealing of the coating by an additional DC polarization of the oxide layer in the original PEO electrolyte solution. 8-HQ acts here as a model inhibitor molecule because it is a potent complexant for both magnesium [12,21] and aluminum [22,23] which are present in the alloy. This inhibitor forms complexes in the form of M*^n^*^+^(8-HQ)*_n_* [22]. The obtained coating systems were characterized in terms of their surface morphology, structure, thickness, roughness, hydrophobicity, chemical composition, and corrosion resistance.

## 2. Materials and Methods

### 2.1. Specimens Preparation and Pre-Treatment

In this research, a 1 mm-thick wrought aluminum alloy sheet of the 6061-T651 variety (Alfun A.s., Bruntál, Czech Republic) was used for the preparation of samples. According to the ASTM B308/B308M-20 standard [24] the composition of the alloy is as follows: 0.8–1.2% Mg, 0.4–0.8% Si, 0.0–0.7% Fe, 0.15–0.40% Cu, 0.04–0.35% Cr, 0.0–0.25% Zn, 0.0–0.25% Ti, 0.0–0.15% Mn, balance Al. The sheet was cut into rectangular samples of dimensions 33 mm × 10 mm or 50 mm × 10 mm. Then, the aluminum alloy specimens were subjected to grinding with water-proof SiC abrasive paper (#400) (Metkon Instruments Inc., Bursa, Turkey) and degreased in a 1:1 deionized water:isopropanol mixture under ultrasonication (5 min). After the samples were dried in the open air, they were sealed by the use of silicon rubber tape such that the non-insulated surface area was equal to either 2.25 cm^2^ (Sample A) or 4.5 cm^2^ (Sample B).

### 2.2. Surface Treatment Protocols

**Figure 1:**
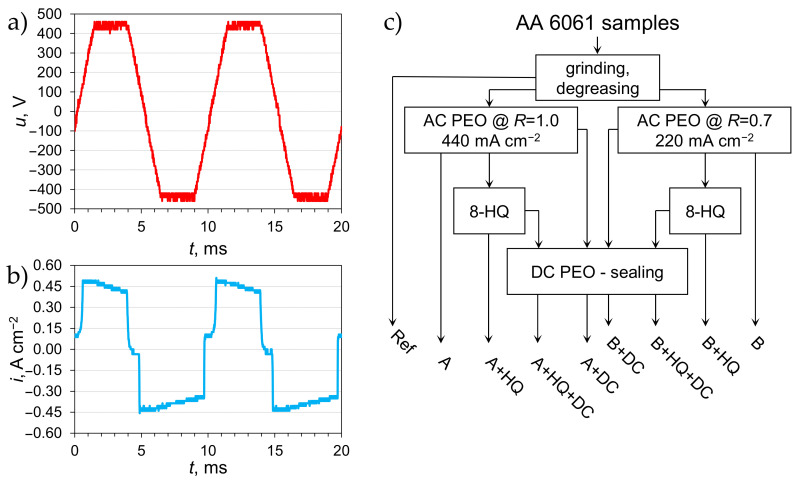
Transient plots (**a**,**b**) showing voltage (**a**) and current density (**b**) waveforms used for the AC plasma electrolytic oxidation (PEO) treatment of aluminum alloy samples and a flowchart visualizing the sample labeling method adopted in the research (**c**).

A few surface modification steps were investigated in this study. First, the samples were subjected to the AC PEO treatment in a solution that contained 0.1 M Na_2_SiO_3_ and 0.05 M KOH (POCH, Avantor^TM^ Performance Materials, Gliwice, Poland). A PCR-1000LE (1 kVA, Kikusui, Yokohama, Japan) AC + DC power supply was utilized for this purpose. The voltage signal during the treatment constituted of a trapezoid waveform of the amplitude of 424 V (the hardware maximum) at the frequency of 50 Hz (Figure 1a). The electrical characteristics of the process were monitored by an oscilloscope (GWInstek GDS-1102, Taipei, Taiwan) with current and voltage probes as well as from the readings of the operating power supply.

Two different current limitations, that were found to produce good quality coatings in the prior research, were imposed in this investigation:

- A: maximum positive peak current density equal to 440 mA cm^−2^ and *R* = 1.0;
- B: maximum positive peak current density equal to 220 mA cm^−2^ and *R* = 0.7.

In this study, *R* is the ratio of the maximum positive peak current density to the maximum negative current density. While the process was under current control the typical current waveform was similar to that in Figure 1b. The oxidation time for both sample types was set to 1 h.

The PEO process was run in a 1-L glass electrolyzer (VWR International LLC, Radnor, PA, USA). The heat of the treatment was absorbed by the use of a cryostat that was circulating liquid at ca. 10 °C in the cooling jacket of the electrolysis cell. Intense mixing of the electrolyte was provided thanks to a magnetic stirrer. The aluminum alloy samples constituted the anode, while the cathode was made of stainless steel.

After the AC PEO treatment, the samples were cleaned in an intense stream of tap water, followed by thorough rinsing with deionized water.

Next, they were put to a laboratory dryer at 60 °C for 24 h (samples A and B in Figure 1c). Then, the surface of the samples could be modified further by one of three routes (Figure 1c):

- 8-HQ: The surface of a dried AC PEO coating was impregnated with a 1 g L^−1^ ethanolic solution of 8-hydroxyquinoline (8-HQ, ACS reagent, ACROS Organics BVBA, Geel, Belgium) by the use of an automatic pipette; the amount of solution dosed on the modified surface was 17.78 μL cm^−2^; the solvent was then allowed to evaporate in the open air for at least 15 min (samples “+HQ”);
- DC: After drying the sample was mounted again in the electrolytic cell (the same electrolyte as in the AC PEO process) and subjected to the DC polarization treatment by incrementally increasing cell voltage from 0 to 400 V for 60 s (voltage ramp) followed by keeping the voltage at 400 V for the next 120 s; after this step was done, the sample was cleaned with tap and deionized water, then transferred to the laboratory dryer at 60 °C for 24 h (samples “+DC”);
- 8-HQ followed by DC: A sequential combination of the two steps above, where after ethanol had evaporated from the 8-HQ impregnation stage, the sample was subjected to the DC PEO treatment and was then dried at 60 °C for 24 h (samples “+HQ + DC”).

### 2.3. Surface Characterization

The surface morphology of the formed anodic oxide films was investigated using a scanning electron microscope (SEM, TM3000, Hitachi, Tokyo, Japan). The cross-sectional views of the oxide coatings were obtained by mounting the samples in an acrylic resin which was followed by grinding (#240) and polishing (9 μm monocrystalline diamond suspension). The specimens were then transferred to another SEM (Phenom ProX, Phenom-World BV, Eindhoven, the Netherlands), equipped with energy-dispersive X-ray spectroscopy (EDS) system, for the inspection of coatings’ structure, thickness, and elemental composition. Both SEM systems were operated at an accelerating voltage of 15 kV.

The roughness of the modified aluminum alloy surfaces was measured using a contact profilometer (Surftest SJ-301, Mitutoyo, Kawasaki, Japan). Three parallel, equally spaced profiles were collected from each sample. The profiles were 4 mm long. From the profiles, the standard roughness parameters, Ra and Rz, were calculated according to [25].

Contact angle measurements were made using a video-based goniometer (OCA 15EC, DataPhysics Instruments GmbH, Filderstadt, Germany). The investigations were run using 5.0 μL droplets of deionized water in a dynamic contact angle mode. The results constitute an average of at least 3 measurements per type of surface. The time of the measurement was set to 5 min or until a droplet has disappeared.

The phase composition of the samples was identified using Seiffert 3003TT X-ray diffractometer (XRD, RICH. SEIFERT & Co. GmbH & Co. KG, Ahrensburg, Germany) using a copper X-ray tube (k_λ1_ = 1.540598 Å, k_λ2_ = 1.544426 Å, k_β_ = 1.39225 Å). The measurements were made in the range of 2*θ* angle from 5 to 80°.

Experimental samples of Raman spectra were recorded using a Raman microscope (inVia Renishaw, Gloucestershire, UK) equipped with a charge-coupled device (CCD) detector, as well as red (633 nm) and green (514 nm) laser light sources. Calibration was performed with a silicon (Si) calibration sample before the measurements, which were carried out using an Olympus LMPlanFl 50× magnification lens (Olympus Corporation, Tokyo, Japan), with a 5 s acquisition time over the 150–1800 cm^−1^ range, and 10 accumulations per spectrum.

X-ray photoelectron spectroscopy (XPS) investigations were done with a PREVAC EA15 hemispherical electron energy analyzer, 2D multi-channel plate detector, and Al-Kα X-ray source (PREVAC dual-anode XR-40B, Rogów, Poland) with the energy of 1486.6 eV (PREVAC Sp. z.o.o., Rogów, Poland). The base pressure was equal to 7 × 10^−9^ Pa. The spectra were acquired with pass energy equal to 200 eV and scanning step equal to 0.9 eV for survey scan, and for the high-resolution spectra: 100 eV pass energy and 0.05 eV scanning step. All spectra were recorded with a normal take-off angle. The binding energy scale was calibrated with respect to the C–C component of C1s spectra (284.8 eV) [26]. The acquired spectra were fitted using CASA XPS^®^ software (version 2.3.23). Shirley function was used for background subtraction, while the components were represented with Gaussian (70%) and Lorentzian (30%) lines.

### 2.4. Electrochemical Corrosion Resistance Measurements

Electrochemical experiments were carried out in a 250 mL glass cell filled with a naturally aerated 0.1 M solution of NaCl at 25 °C. A three-electrode configuration was adopted for the studies. It constituted a working electrode (the sample to be measured), a saturated calomel electrode (SCE) filling the role of the reference electrode, and a platinum mesh counter-electrode. The measurements were operated using a potentiostat-galvanostat (PARSTAT 4000, Princeton Applied Research, Ametek, Berwyn, PA, USA) with a dedicated VersaStudio software for data acquisition and processing. Prior to the polarization studies, the samples were stabilized in the corrosion medium for 1 h. Next, the potentiodynamic polarization (PDP) curves were recorded in the potential range from −300 mV vs. open-circuit potential (OCP) to +300 mV vs. SCE. After reaching the apex potential, the polarization direction was reversed to see if the coating has undergone oxide breakdown. In some cases, the point at which the polarization was reversed was chosen based on the sharp current density peaks in the curve, marking the breakdown phenomenon. The scan rate of the PDP experiments was set to 10 mV min^−1^. The experiments for each of the investigated surfaces were run in triplicate.

## 3. Results and Discussion

### 3.1. Formation of AC PEO Coatings on the AA 6061 Samples

**Figure 2:**
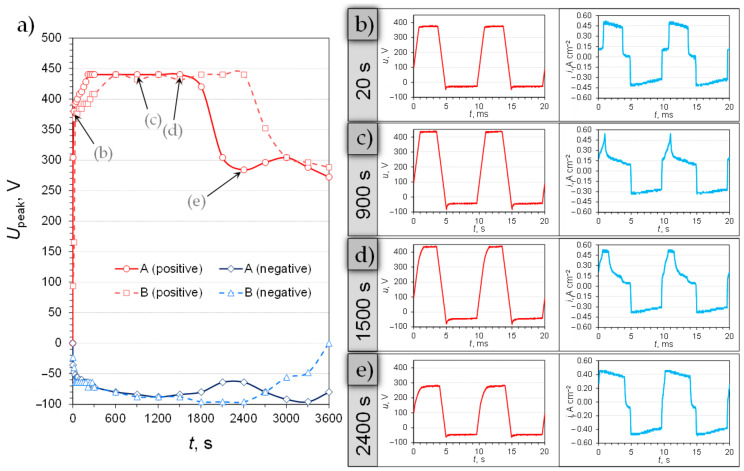
Voltage-time diagram (**a**) depicting positive and negative peak voltage changes during AC PEO of the aluminum alloy samples at 440 mA cm^−2^ (A) and 220 mA cm^−2^ (B). Transient plots (**b**–**e**) show the voltage and current density waveforms recorded at 20 s (**b**), 900 s (**c**), 1500 s (**d**), and 2400 s (**e**) after the beginning of the process run at 440 mA cm^−2^ (A).

Surface treatment of the 6061 aluminum alloy comprised of some steps (as summarized in Figure 1c). The first of those steps was AC PEO and the course of this treatment is presented in Figure 2a. The plot shows the comparison between two of the studied variants of AC PEO, i.e., at the higher peak current density and symmetrical peak current limitations (*R* = 1.0) and at lower peak current density and asymmetrical current signal (the negative current limit higher than the positive one; *R* = 0.7). The selected treatment conditions were chosen because “soft-sparking” conditions could be observed relatively early in the process. In the case of the A process, a sharp drop in the cell voltage occurred at ca. 1800 s, while for the B variant of the AC PEO it was close to 2400 s.

As it was shown by Rogov et al. [10], the moment of transition to the “soft-sparking” stage is incidental with the preferential formation of *α*-Al_2_O_3_ phase nearby the metal-oxide interface. For this process to begin a significant thickness of *γ*-Al_2_O_3_-rich oxide coating must be present on top of the substrate. Only then the heat transfer from the oxide to the electrolyte is hampered enough to allow for the high-temperature corundum generation and “soft sparking” to begin. Figure 1b–e shows how the voltage and current transients evolved during the processing of the A sample. At the beginning of the anodic oxidation, the process was controlled by current and the cell voltage was increasing linearly with time (Figure 2a,b). The onset of sparking was found to be at approximately +370 V for both types of samples. Once the positive peak cell voltage reached the hardware limitation (+424 V), less and less electric charge was passed in the anodic half-cycle of the treatment (Figure 2a,c). It can be explained by the rising resistance of the formed coating which could not be surpassed by the limited voltage. At the same time, the process was run at the limiting current density in the negative half-cycle (cell voltage of approximately −80 V). Consequently, a necessary asymmetry between the positive and negative charges passed through the system arose [5,9,10]. It can be noted that at 1500 s (Figure 2a,d) the quality of the voltage and current transients changed, as the time spent at the current limitation was growing in proportion with respect to the voltage limitation period. Therefore, it can be noted that at this point the coating was beginning to become more conductive for the passage of the anodic charge which is a characteristic of the nearing onset of “soft-sparking”. The transients at 2400 s (Figure 2a,e) show that the treatment was then conducted under current control and at relatively low anodic and cathodic cell voltages (ca. +300 and −65 V, respectively). Once the voltage dropped, a typical silencing of surface sparks was observed.

**Figure 3:**
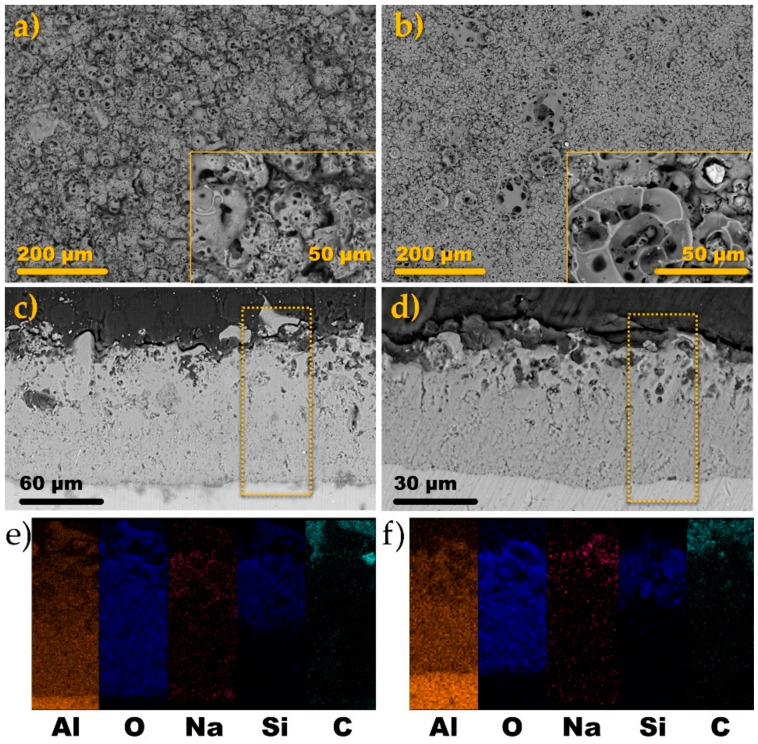
Planar (**a**,**b**) and cross-sectional (**c**,**d**) SEM images of the anodic oxide films formed on 6061 aluminum alloy substrate under the conditions A (**a**,**c**) and B (**b**,**d**). EDS mapping analysis of the dotted-line rectangles in the cross-sections of the coating A (**c**) and B (**d**) are presented in (**e**) and (**f**), respectively.

Surface morphologies of the coatings produced via AC PEO treatment can be inspected from Figure 3a,b. It is immediately noticeable that the coating formed at the higher current density (A) has a much more rugged, sponge-like appearance (Figure 3a). In fact, it is sometimes practiced to remove this topmost layer by polishing because it exhibits much poorer mechanical properties than the underlying, compact oxide [27].

In the case of the B sample (Figure 3b), the surface is much smoother and it displays some point-type, globular structures. These structures were formed probably due to the encapsulation of the gas bubbles in a growing oxide (which explains why most of them are cracked or half-open). Figure 3c,d presents the cross-sectional views of the coatings A and B, respectively. From the SEM images, it can be told that the anodic oxide films are relatively uniform in thickness and are composed of two sublayers, i.e., outer porous and inner compact sublayers. This is a typical PEO oxide coating structure [4,5]. It was found that on average the A coating was 2–3 times thicker than the B coating (Table 1).

Interestingly, it can be noted that the B coating, formed at half of the current density of the A process, exhibited the compact layer thickness of 18.6 μm which was ca. 50% of that of the A coating (39.4 μm). Thus, it has been shown that the process conducted at the lower current density was more effective at forming the compact oxide sublayer [28]. In the case of both of the coatings, the EDS analysis revealed they were composed of Al, O, Na, and Si. Aluminum and oxygen were uniformly dispersed throughout the layers, while sodium and silicon were concentrated in the outer, more porous part of the coating. Such findings were also reported by others [29,30,31].

### 3.2. Further Surface Modifications of the PEO Oxide Films

**Figure 4:**
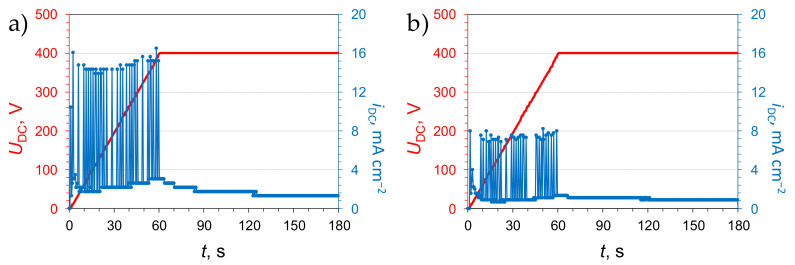
Voltage and current density transients recorded during DC PEO sealing treatment after the impregnation of the anodic oxide layers, produced in the A (**a**) and B (**b**) AC PEO procedures, with 8-hydroxyquinoline.

The coatings after drying were subjected to additional treatment methodologies. The oxide films formed in this study were designed to have a highly-porous, extensive surface area (Figure 3) to allow for the rapid distribution of the impregnation liquid through the capillary action. Therefore, the first step of the modification of the PEO films with a corrosion inhibitor, 8-hydroxyquinoline, was the dropwise dosing of the 1 g L^−1^ ethanolic solution of 8-HQ on its surface. After the solvent was fully evaporated, the surface of the coating attained a greenish-yellow tint. To ensure that the inhibitor was not easily washed out from the loaded PEO coating, an attempt to seal the coating with an additional electrochemical treatment was made. It encompassed mounting the inhibitor-impregnated film (the “+HQ” sample) in the electrolytic cell filled with the 0.1 M Na_2_SiO_3_ + 0.05 M KOH solution (the same as for the AC PEO treatment). Then, a DC voltage was supplied to the sample as it was anodically polarized to the voltage of 400 V in a gradual manner (Figure 4).

Consequently, a current was observed to flow. During the voltage ramp, current spikes were observed. It was reasoned that they might have originated either from the anodic destruction of the inhibitor or ongoing passivation of the oxide progressively filled with the electrolyte. When the final voltage was attained, the current was found to decay. The intensity of the current peaks observed in the case of the B series samples (Figure 4b) was half of that recorded for the A series samples (Figure 4a). The reason for that is due to the difference in the geometric surface area of the samples which was twice as high for the B series coatings. Therefore, the absolute current intensity of the peaks was comparable for both of the sample series. It is noteworthy, that during the DC sealing treatment very fine, inaudible and relatively few sparks could be observed on the polarized surfaces. Therefore, it might be hypothesized that a mild PEO process took place.

### 3.3. Surface Characterization of the Modified PEO Coatings

**Figure 5:**
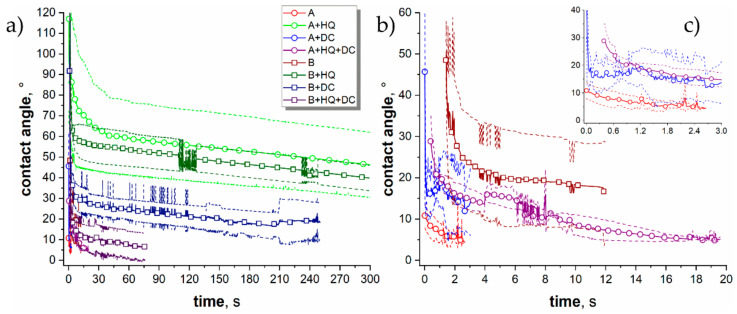
Contact angle vs. time variations presented at three different magnifications (**a**–**c**) recorded for the PEO coatings prepared under different processing conditions. Solid lines with symbols correspond to the average values whereas the dashed lines of the same color refer to the respective standard deviations.

To point out which of the preparation steps affected the surface properties of the resulting coating systems in a meaningful way, a series of experiments have been undertaken. The surface roughness measurements results are summarized in Table 2. The contact angle vs. time variations are presented in Figure 5. As it was evident from the SEM images (Figure 3), the surface of the A sample was much rougher than that of the B sample. Both of the roughness parameters (Ra and Rz) were approximately 3 times larger for the A variant of the coating, compared to the B sample. The highly porous oxide layers were so effective at soaking in the moisture that the measurement of the contact angle was impossible for the neat A and B samples in the static contact angle mode.

It is why the dynamic measurements were attempted. The results of the experiments can be inspected in Figure 5.

**Figure 6:**
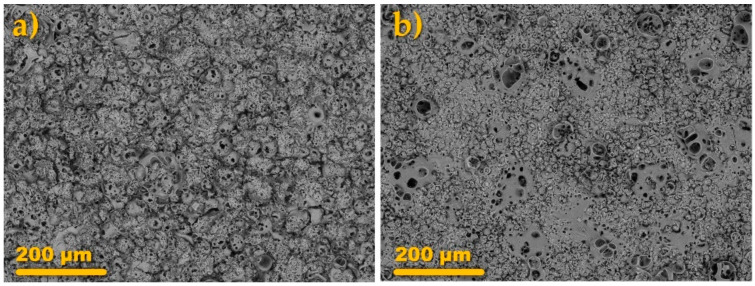
SEM planar images of the A + HQ + DC (**a**) and B + HQ + DC (**b**) samples.

The droplets were soaked into the coating A (via spreading of the liquid and filling of the pores) very rapidly, and after 2–3 s, the angle was impossible to measure by the software (Figure 5c). A similar situation was encountered for the B coating where the soaking time was approximately 12 s, however, in such a short period of the measurements the errors between the parallel samples were comparatively high. The studies showed that the impregnation of the AC PEO coatings (A and B) with 8-HQ did not change the surface roughness of the samples (A + HQ and B + HQ) in a statistically significant manner. The provision of 8-HQ via impregnation led to the significant increase of the contact angle (Figure 5a) and the droplets remained relatively stable on the surface throughout the entire measurement. Slightly higher average values (approximately 50 vs. 45°) were recorded for the A-HQ sample as compared with the B-HQ surface, however, the differences were insignificant. It could be owed to the formation of an organic film of the inhibitor which repelled water from entering the surface pores of the coating. It was demonstrated that the modification of alumina with 8-HQ may indeed improve the water-repellent properties of a material [32]. An additional DC sealing treatment brought about a change in both surface roughness and surface energy (Table 3, Figure 5a,b). In the case of the A + HQ + DC sample, the surface roughness (as represented by Ra parameter) measured using a contact profilometer decreased from 9.26 ± 1.16 down to 6.63 ± 0.37. Interestingly, the opposite trend was observed for the B series analog where, after the sealing, the Ra parameter increased from 2.50 ± 0.42 down to 4.45 ± 0.25. Taking into account that the modification of the original AC PEO coatings did not change the SEM surface morphology of the resulting A + HQ + DC sample in an appreciable way (Figure 6a), it has to be concluded that the changes in the roughness, as measured with a contact profilometer, must be due to filling in of the pores with the oxidation products. Whereas the increase of the roughness encountered for the B + HQ + DC sample was caused by the formation of additional, globular structures (Figure 6b). The similar surface features, yet less numerous, were spotted on the neat B coating surface (Figure 3b) as well. What the A + HQ + DC and B + HQ + DC samples had in common was the increase of the contact angle in relation to the neat coating (Figure 5a,b).

Although the contact angles were not as high as in the case of the 8-HQ impregnated coatings (Figure 5a) and the effectiveness of the three-step coating was much better in the case of the B + HQ + DC sample (time until the disappearance was ca. 70 s as compared with 20 s for A + HQ + DC). This result should be rationalized as a combined effect of hydrophobization due to the organic phase film formation as well as the partial filling of the pores by the oxidation products (be it organic or inorganic in origin). The additional DC treatment could eradicate the surface of the coatings from the adsorbed 8-HQ film while still maintaining some of it in the pores. To prove that the 8-HQ impregnation had any effect on these surface characteristics of the coatings after DC PEO sealing, a supplementary series of samples comprising AC PEO treatment followed by drying and additional DC oxidation was prepared (“+DC” samples). Such a combination of treatments yielded surfaces that were more (B series) or less (A series) rough than the neat coatings (Table 2). They were also more hydrophobic than the non-modified AC PEO oxide films, irrespective of the original coating type. However, they were not as hydrophobic as the combined HQ and DC or HQ variants, evidencing the meaningfulness of the inhibitor impregnation step.

**Figure 7:**
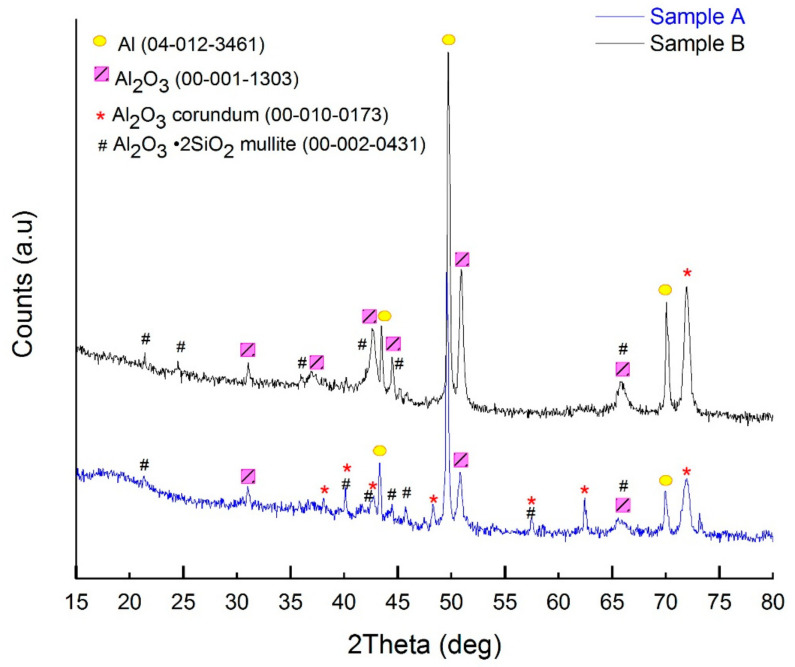
XRD pattern of the anodic oxide films formed on 6061 aluminum alloy surface under the treatment conditions A and B. PDF ref. cards of the corresponding phases are included in the parentheses.

XRD was utilized to investigate the phase composition of the anodic oxide films and the results of the analysis of the neat A and B coatings are presented in Figure 7.

The diffraction patterns corresponding to the modified oxide coatings (with 8-HQ and DC PEO steps) gave inconclusive results (no noticeable additional reflexes), which is why the authors decided not to include them in the study. From the patterns in Figure 7, it can be said that the coatings contained both crystalline and non-crystalline phases (amorphous halo between 2*θ* angle of 15 and 20° [13]). Apart from the signals originating from the metal substrate (Al—04-012-3461), there are signals corresponding to aluminum oxide and mixed aluminum and silicon oxide phases. Corundum (*α*-alumina 00-010-0173) was identified only in the A sample, while *γ*-alumina (00-001-1303) and mullite (Al_2_O_3_∙SiO_2_) were found in both of the coatings. It is noteworthy that corundum, a hard and durable aluminum oxide phase, is formed preferentially under the “soft-sparking” regime [9,10] which was determined to last longer during the preparation of the A samples (Figure 2a) as compared with the B samples. As the phase composition of A was more satisfactory than that of B, further surface characterization studies were limited to the former series of coatings.

**Figure 8:**
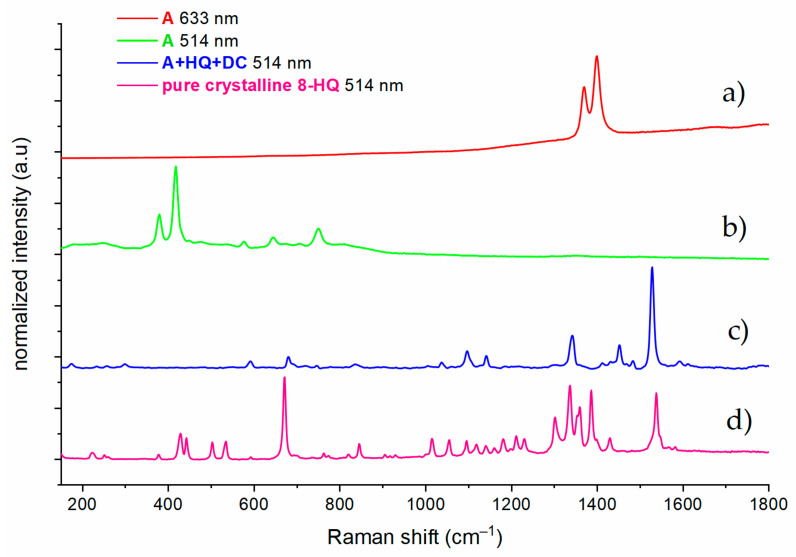
Raman spectra of the anodic oxide layers before (**a**,**b**) and after (**c**) the post-treatment procedures (impregnation with 8-hydroxyquinoline followed by DC PEO sealing) recorded at different laser excitation wavelengths (633 or 514 nm); neat 8-HQ spectrum (**d**) is provided for comparison.

Figure 8a,b presents the characteristic spectra for the A samples not impregnated with HQ.

The recorded spectrum corresponds to *α*-Al_2_O_3_ with a spinel structure. Based upon D^6^_3d_ symmetry of *α*-Al_2_O_3_, seven Raman active phonon modes, 2A_1g_ + 5E_g_, have been reported. Six signals were recorded on the spectra of the samples at 378, 417, 430, 577, 645, and 750 cm^–1^. This is consistent with literature data [33]. A raised baseline indicates the possible presence of other forms of Al_2_O_3_ which is in accord with XRD data (Figure 7). The broadening of the signal proves its low degree of crystallinity. When the spectrum was recorded with excitation provided by a 633 nm laser light irradiation, the dominant signals became the peaks at 1350 and 1450 cm^−1^ (Figure 8a). These signals are the effect of fluorescence induced from Fe^3+^ ions from the alloy substrate that replace Al^3+^ ions in the spinel structure, which additionally confirms that *α*-Al_2_O_3_ is the dominant structure [34].

In the case of the A + HQ + DC samples, spectra with signals originating from 8-HQ and its degradation products were recorded (Figure 8c). The neat 8-HQ spectrum (Figure 8d) is also shown for comparison. The A + HQ + DC spectrum shows only the most intense 8-HQ signals at 1537, 1337, and 671 cm^−1^. However, additional signals appear in the spectrum at 1483, 1452, 592, 296 cm^−1^. Peaks at 1483 and 1452 cm^−1^ correspond to the formation of carbon-carbon bonds, accompanying the degradation of organic compounds (or soot formation [35]). These results support the claim that the superficially-adsorbed 8-HQ was destroyed in the course of the additional DC treatment, however, some of it (or its degradation products) could still remain in the pores. It would explain the longer penetration of the water droplets into the pores (Figure 5).

**Figure 9:**
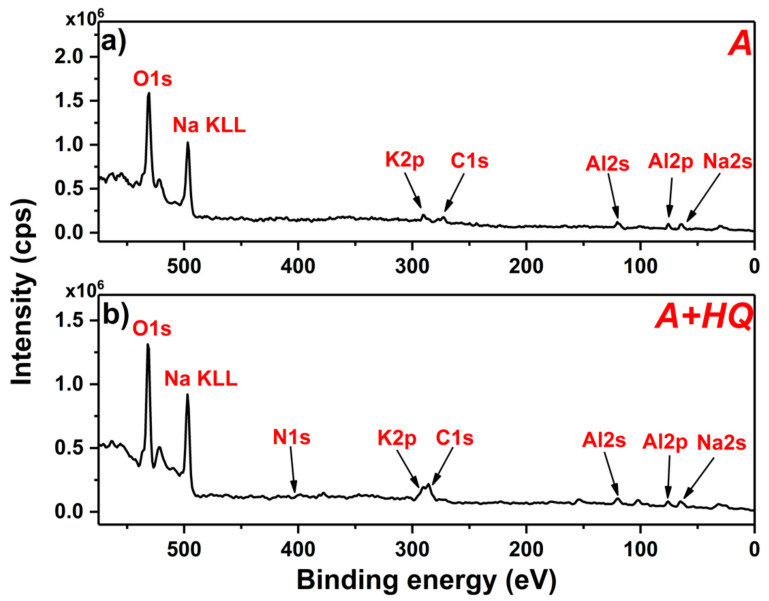
XPS survey spectra recorded for the A (**a**) and A + HQ (**b**) samples.

The chemical composition of the A, A + HQ, and A + HQ + DC coatings was investigated by the XPS technique. The wide scan spectra recorded for A and A + HQ (Figure 9) reveal the presence of Al2p and O1s signals at ca. 75 eV and 530 eV, respectively, being specific for Al covered with an oxide layer, and from the species coming from the plasma electrolytic oxidation bath, i.e., potassium and sodium.

Additionally, for both samples C1s signal is observed at ca. 285 eV, which comes from the organic inhibitor and/or adventitious carbon [26,36]. Taking into account the high-resolution C1s and Al2p regions after correcting the signal intensity for the element and transition specific photoemission cross-sections, the surface atomic ratios of C:Al were calculated (Table 3).

**Figure 10:**
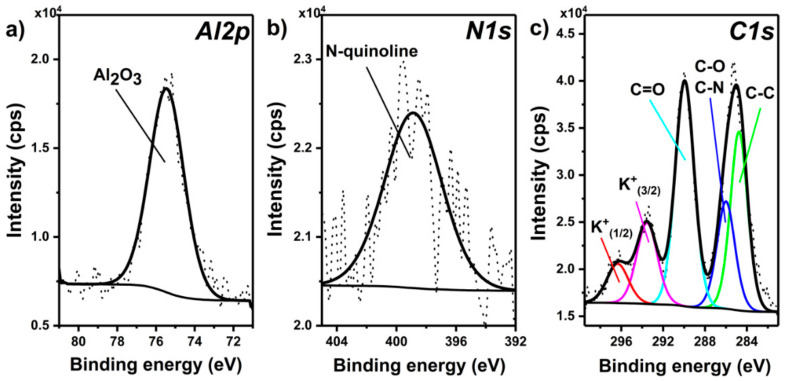
High-resolution XPS spectra of Al2p (**a**), N1s (**b**) and C1s and K2p (**c**) recorded for the A + HQ sample.

The significant increase in the carbon-content for the A + HQ sample can be attributed to the deposition of the organic inhibitor on the surface. The presence of 8-hydroxyquinoline in A + HQ is further confirmed by the occurrence of N1s signal at ca. 399 eV [37,38]. It is worth noting that the C:Al atomic ratio after the DC PEO sealing treatment went back to more or less original proportion. It might mean that virtually all of the inhibitor adsorbed on the outer surface of the oxide has been destroyed in the course of anodic oxidation. It is in accord with the contact angle (Figure 5) and Raman (Figure 8) results. In the next step, high-resolution spectra were recorded for the A and A + HQ samples, giving more details about the chemical structure of the formed oxide film. The high-resolution XPS spectrum of Al2p region recorded for the A + HQ sample (Figure 10a) reveals the presence of one asymmetric component at 75.4 eV, which is characteristic for Al_2_O_3_ formed on Al substrate [39].

Importantly, no signal coming from Al metal is observed at 72.8 eV which indicates the good homogeneity of the oxide layer. A similar result was observed for A, confirming that Al_2_O_3_ was formed in the AC PEO process. As shown in Figure 10b, in the case of the N1s region, only one component is observed at 398.9 eV, which can be assigned to the nitrogen present in the 8-hydroxyquinoline ring [37,38]. Finally, the decomposition of the high-resolution spectrum of the overlapping C1s and K2p regions reveals the presence of five signals with maxima at 284.8, 286.0, 289.9, 293.5, and 296.3 eV that can be assigned to C–C, C–O/C–N, C=O components of C1s and K^+^(2p3/2) and its spin-orbit splitting counterpart, respectively (Figure 10c). While C–C and C–O/C–N can be assigned to both—the organic layer and the adventitious carbon residues, the C=O arises only due to the latter. Similar components are present in the C1s region of the A sample, though the relative intensity of the C=O component is significantly higher when compared to the C–C component (Table 4), which confirms the presence of the organic layer on the A + HQ surface.

### 3.4. Electrochemical Corrosion Resistance Measurements

**Figure 11:**
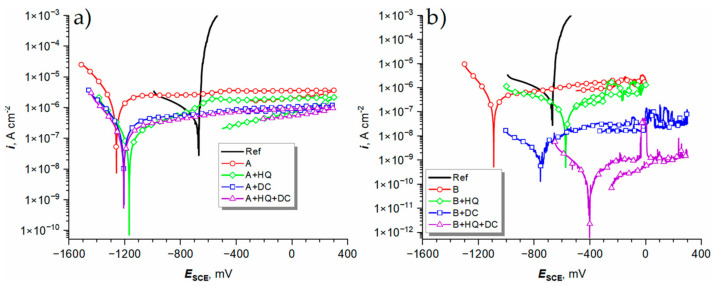
Potentiodynamic polarization curves recorded for the anodic oxide films formed under the process conditions A (**a**) and B (**b**), and for the samples which were additionally impregnated with 8-HQ (+HQ), DC PEO-ed (+DC), or both impregnated with the inhibitor and DC PEO-ed (+HQ + DC), after 1 h of immersion in 0.1 M NaCl solution at 25 °C.

The final test to the proposed coating systems was their exposition to the model corrosion medium—a 0.1 M NaCl solution. After 1 h of OCP stabilization in the medium PDP experiments were commenced. The results are shown in Figure 11. A group of selected corrosion resistance parameters, such as corrosion potential (*E*_cor_), passive oxide breakdown potential (*E*_b_), and passivation current density at the apex potential (or just before the onset of the oxide breakdown; *i*_pas_), was extracted from the gathered data (Table 5).

The PDP curve corresponding to the aluminum alloy substrate (Ref) displayed typical features of a metal surface corroding with the formation of pits in the oxide layer, through which the metal is actively dissolved. Therefore, in the case of Ref, *E*_cor_ is the same as *E*_b_ (Table 5). The relatively flat cathodic branch of the curve (Tafel slope, *β*_c_, equal to ca. −0.5 V dec^−1^) suggests that the overall rate of the process is limited by the oxygen diffusion to the surface of the alloy. *E*_cor_ of the A series samples (Figure 11a, Table 5) is equal to ca. −1250 mV vs. SCE which is markedly lower than that of Ref. However, Tafel slopes were much steeper in this case (*β*_c_ ≈ +0.2 V dec^−1^), meaning that oxygen diffusion had a markedly less impact on the kinetics of corrosion. This also led to the development of much more negative values of *E*_cor_ (closer to the standard potential of aluminum; −1574 mV vs. SCE). Nevertheless, none of the A series coatings underwent breakdown when polarized up to the potential of +300 mV vs. SCE. Moreover, it was found that the impregnation of the oxide film with 8-HQ decreased *i*_pas_ of the A + HQ sample as compared with the neat A coating (Figure 11a, Table 5). An even more pronounced effect was noted for the A + DC sample, which means that the DC PEO passivation (after drying) alone is a potent method of boosting the corrosion resistance of the AC PEO coatings. Inconclusive results were obtained for the A + HQ + DC samples, which were characterized by poor reproducibility (high standard deviation in Table 5). It might suggest that the DC sealing of the coating in the presence of 8-HQ was non-uniform in character. A similar result of only a slight improvement of the tightness of the A + HQ + DC surfaces as compared with the A + DC sample was encountered in the contact angle measurements (Figure 5). Much better reproducibility in that regard was encountered for the B series samples (Figure 11b, Table 5). In the case of the B sample, *E*_cor_ (−1175 mV vs. SCE) was similar to that of the A series specimens. However, two out of three of these samples were found to undergo breakdown at ca. −270 mV vs. SCE. This finding is in contrast with the superior breakdown resistance of the A series samples which were much thicker than their B counterparts. It was determined that for the B series specimens, the adopted methods of improving the corrosion resistance of the PEO-ed aluminum alloy substrate all brought about satisfying effects. *E*_cor_ was significantly shifted towards higher potentials. In the case of the B + HQ sample, the oxide breakdown was observed at −99 ± 43 mV vs. SCE, which was also higher than that of the neat B oxide films. Moreover, even after noticing the breakdown phenomenon, the provision of the corrosion inhibitor led to the healing of the breach and after the reverse polarization, the current density dropped below the forward polarization curve, proving the beneficial effect of 8-HQ. The additional DC passivation of the B coating (B + DC) was even more effective, with the best results obtained for the B + HQ + DC sample, where the *i*_pas_ was reduced by three orders of magnitude as compared with the neat B oxide film. Although it has to be said that the problem of relatively poor reproducibility of the results (one sample out of three suffered from the oxide breakdown at 38 mV vs. SCE) was encountered also in this series of samples. Interestingly, the best corrosion resistance was not determined for the “+HQ” samples, although the highest contact angles were recorded for these sample series (Figure 5). It could mean that the inhibitor could be easily desorbed from the treated surfaces and only the additional DC treatment can improve the corrosion resistance by filling the pores with corrosion or 8-HQ degradation products. However, more research on this promising PEO oxide films surface sealing method is required.

## 4. Conclusions

From the conducted experiments the following concluding remarks can be drawn:

- The PEO coatings were capable of rapidly soaking in the solution of 8-hydroxyquinoline in ethanol which served to uniformly distribute the substance throughout the oxide that was then allowed to evaporate leaving the surface enriched with the corrosion inhibitor;
- The provision of the inhibitor to the oxide layers led to the slight surface hydrophobization without influencing the surface roughness and morphology of the original coating and improved the corrosion resistance of the coating system;
- The high-voltage DC treatment of the inhibitor-impregnated oxide films gave rise to the destruction of the superficially adsorbed inhibitor as evidenced by the XPS studies;
- At the same time, the DC PEO sealing treatment allowed for filling of the pores with the oxidation products (some of it was the inhibitor degradation products, possibly soot), as it was found from the Raman investigations;
- As a result, the complete HQ + DC treatment allowed for providing superior corrosion resistance of the 6061 aluminum alloy in a NaCl solution;
- The better results of the corrosion inhibition were found for the thinner of the two coating systems (coating B), probably due to the lower porosity of the original coatings which were filled by the inhibitor more readily and allowed for easier sealing under the DC conditions;
- The fact that DC PEO sealing alone did not bring about the same anti-corrosion properties as the combined 8-HQ impregnation and DC sealing treatment dispels the notion that the provision of the inhibitor was a needless step in the procedure.

Nonetheless, the adopted method of surface treatment of the aluminum alloy is still plagued by the problem of unsatisfactory uniformity of the sealing for the thicker coatings. This needs to be amended in future efforts for optimization of various steps of the procedure.

## Figures and Tables

**Table 1:** Thicknesses of the anodic oxide films on the 6061 aluminum alloy substrate formed under the AC PEO conditions A and B.

Sample Label	Total Thickness, μm	Compact Layer Thickness ^1^, μm	Compact to Total Layer Thickness
A	107.9 ± 22.9	39.4 ± 6.3	36.5%
B	37.7 ± 9.0	18.6 ± 2.5	49.3%

^1^ estimated based on the porosity difference of the highly-porous and compact oxide sublayers.

**Table 2:** Surface roughness measurements results of the 6061 aluminum alloy samples subjected to different surface finishing procedures.

Sample Label	Ra, μm	Rz, μm
A	8.66 ± 0.08	48.5 ± 1.0
A + HQ	9.26 ± 1.16	50.7 ± 3.8
A + DC	7.19 ± 0.20	41.9 ± 3.2
A + HQ + DC	6.63 ± 0.37	39.4 ± 1.2
B	2.94 ± 0.19	18.5 ± 1.1
B + HQ	2.50 ± 0.42	16.3 ± 2.1
B + DC	3.30 ± 0.07	20.7 ± 0.9
B + HQ + DC	4.45 ± 0.25	27.3 ± 1.4

**Table 3:** Surface atomic ratios measured by XPS for the A and A+ HQ samples.

Sample Label	C:Al
A	1:0.63
A + HQ	1:0.40
A + HQ + DC	1:0.64

**Table 4:** The ratio of the components of C1s region for the A and A+ HQ samples.

Sample Label	Ratio
A	C–C:C–O:C=O = 1:0.18:2.27
A + HQ	C–C:C–O/C–N:C=O = 1:0.63:1.37

**Table 5:** Selected corrosion parameters obtained from the analysis of potentiodynamic polarization (PDP) curves from Figure 11.

Sample Label	*E*_cor_, mV vs. SCE	*E*_b_, mV vs. SCE	*i*_pas_, nA cm^−2^
Ref	−669 ± 1	−669 ± 1 ^3^	−
A	−1283 ± 21	−	4450 ± 850
A + HQ	−1210 ± 40	−	2540 ± 350
A + DC	−1221 ± 13	−	1130 ± 80
A + HQ + DC	−1236 ± 27	−	1710 ± 700
B	−1175 ± 75	−269 ± 105 ^2^	1260 ± 180
B + HQ	−591 ± 15	−99 ± 43 ^3^	360 ± 80
B + DC	−673 ± 85	−	28.0 ± 6.1
B + HQ + DC	−493 ± 78	38 ^1^	2.90 ± 1.20

^1^ One out of three samples underwent oxide breakdown; ^2^ two out of three samples underwent oxide breakdown; ^3^ three out of three samples underwent oxide breakdown.

## Data Availability

Data is contained within the article or supplementary material.
